# Sintering Inhibition of Silver Nanoparticle Films via AgCl Nanocrystal Formation

**DOI:** 10.3390/nano7080224

**Published:** 2017-08-17

**Authors:** Thomas Öhlund, Magnus Hummelgård, Håkan Olin

**Affiliations:** Department of Natural Sciences, Mid Sweden University, SE-85170 Sundsvall, Sweden; magnus.hummelgard@miun.se (M.H.); hakan.olin@miun.se (H.O.)

**Keywords:** sintering, chemical sintering, inkjet printing, silver nanoparticles, thin films, flexible substrates, paper coatings, printed electronics, flexible electronics, papers

## Abstract

Electrically conductive films are key components in most printed and flexible electronics applications. For the solution processing of conductive films, inks containing silver nanoparticles (AgNPs) remain important because of their relatively easy processing and generally low resistivity after a sintering procedure. Because the commonly used, moderate sintering temperatures of 150–300 °C are still too high for most low-cost flexible substrates, expanding the knowledge of surface-ink interactions that affect the sintering temperature is desirable. It is known that chloride ions can assist the sintering of AgNP films by displacing capping agents on the surfaces of AgNPs. However, very little is known about other possible Cl-AgNP interactions that affect the resistivity and no interaction having the opposite effect (sintering inhibition) has been identified before. Here we identify such a Cl-AgNP interaction giving sintering inhibition and find that the mechanism involves the formation of AgCl nanocrystals within the AgNP film. The AgCl formation was observed after inkjet-printing of AgNP inks with polyvinylpyrrolidone (PVP) as the capping agent onto papers with quick-absorbing coatings containing 0.3 wt % KCl. Our findings show that chloride can have opposite roles during sintering, either assisting or inhibiting the sintering depending on the prevalence of AgCl formation. The prevalence of AgCl formation depends on the absorption properties and the capping agent.

## 1. Introduction

The solution processing of functional materials and their deposition by various coating and printing methods have established a foundation for flexible and printed electronics. With the paradigm shift from traditional subtractive processing to additive manufacturing, cost savings and environmental advantages can be realized. In almost every electronic device and application, electrically conductive films are crucial. For solution-processed metal films, dispersions (inks) of silver nanoparticles (AgNPs) remain the most common choice because of their commercial availability and processability under ambient conditions and moderate temperatures. To prevent AgNP aggregation before deposition, organic capping agents are attached to the NP surface to form a protective shell. These organic shells induce electrostatic or steric repulsion when NPs approach and organic ligands begin to overlap. After ink deposition, the AgNP film is usually sintered by applying heat to decrease resistivity. The heat melts the polymeric shells, facilitating direct contact between NPs and initiating the fusion of the particles.

Environmentally friendly, low-cost, flexible substrates are desirable, and thus paper-based substrates are an interesting option. The adaptability of papers can be improved by applying suitable coatings to reduce the surface roughness and increase the absorption rate [1,2]. However, the temperature sensitivity of paper and other low-cost flexible substrates is problematic when sintering NP inks using traditional heating. To overcome this issue, alternative sintering methods [3,4,5] have been investigated. Some of the most promising methods rely on intense pulsed light [6,7,8] or chemical sintering agents [9,10,11,12,13].

The presence of chemical sintering agents can lower the sintering temperature by inducing the desorption of the capping agent from the AgNP surface [12]. In particular, compounds releasing chloride ions have been found to substantially lower sintering temperatures, with some even being effective at room temperature. In one study, AgNPs capped with poly(acrylic acid) (PAA) were sintered in room-temperature by introducing poly(diallyldimethylammonium chloride) (PDAC) [12]. The researchers observed the room-temperature sintering when substrates were pretreated with PDAC and when AgNP films were post-treated with PDAC solution. In another study, a small amount of NaCl was added into a AgNP/PAA ink [11]. The NaCl concentration was sufficiently low as to not affect the stability of the ink in the dispersion but, upon drying, the increase in the concentration induced room-temperature sintering. These researchers demonstrated that the sintering was induced by the desorption of the capping polymer by chloride ions replacing the polymer anchoring groups at the AgNP surface. In a third study, paper with active coatings containing a low concentration of KCl induced room-temperature sintering of inkjet-printed AgNP films [13]. During the ink absorption, chloride ions migrated from the coating into the AgNP film, thereby increasing in concentration and inducing sintering.

However, there has been at least one conflicting report that chloride and other halides induced a reverse effect on paper coatings, significantly increasing the AgNP film resistivity [14]. Such increased resistivity is not consistent with the known mechanism of capping agent desorption, hence calling for further investigations. Here, we perform the needed investigation and find a resistivity increase when PVP-capped AgNPs are inkjet-printed on papers containing KCl. By examining the AgNP film nanostructure in detail, we find that the resistivity increase is due to the formation of AgCl nanocrystals on the surfaces of AgNPs. The results show that chloride can have the opposite role as a sintering inhibitor of AgNP films on absorptive media. This new insight is useful for the proper design and implementation of sintering methods for printed electronics.

## 2. Results and Discussion

### 2.1. AgNP Films Were Inkjet-Printed onto Active Papers and Reference Papers

**Figure 1:**
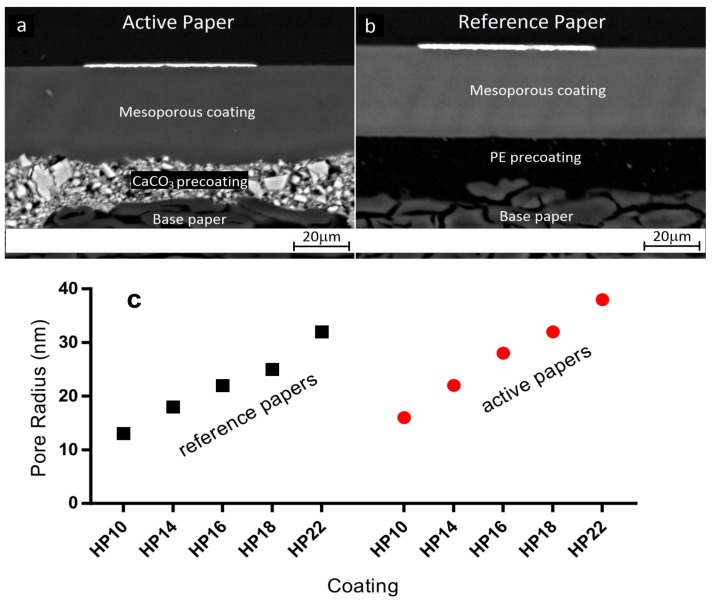
Active papers and reference papers. The construction of the (**a**) active paper series with porous precoating and the (**b**) reference paper series with nonporous precoating (scanning electron microscopy cross-sections). The top white layers are AgNP films. Only the upper part of the base paper is visible. (**c**) Dominant coating pore radius of each paper (mercury porosimetry).

As main substrates, we used two series of custom-made coated papers with varying pore sizes, referred to as active papers and reference papers (Figure 1). Each paper consisted of a base paper, a precoating, and a top coating of mesoporous alumina. The top coatings contained 0.3 wt % of KCl as a sintering agent. The active papers had a porous CaCO_3_ precoating (Figure 1a), whereas the reference papers had a nonporous polyethylene precoating (Figure 1b). Both the active paper series and the reference series were composed of five papers with different top coating pore sizes (Figure 1c). The different coating pore sizes were achieved by using alumina coating pigments with different sizes: HP10, HP14, HP16, HP18 and HP22 (commercial trade names where the number indicates the crystallite size in nanometers). The manufacturing and characterization of active papers and reference papers have been described in detail previously [13].

### 2.2. Resistivity Measurements Showed Sintering Inhibition of AgNP Films on Active Papers

**Figure 2:**
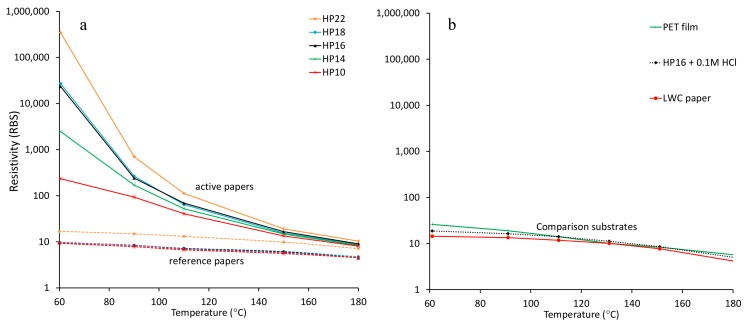
Resistivity of AgNP films showing sintering inhibition on active papers. (**a**) Sintering is inhibited on active papers (solid curves) compared to that on reference papers (dashed curves). (**b**) Pretreating a reference paper with HCl increased the resistivity but the effect was much smaller than the effect on active papers. The resistivities on polyethylene terephthalate (PET) film and lightweight coated (LWC) paper were low compared with that on active papers. Error bars were omitted for visual clarity. RBS = resistivity of bulk silver.

We observed unexpected sintering inhibition for inkjet-printed AgNP films on the active papers. An AgNP ink containing polyvinylpyrrolidone (PVP) as a capping agent and triethylene glycol ethyl ether (TGEE) as a solvent was inkjet-printed onto the active papers, the reference papers, and two commercial substrates: polyethylene terephthalate (PET) film and lightweight coated (LWC) paper. After printing, we oven-sintered the printed films at different temperatures and measured the resistivity after each temperature. The resistivity of the AgNP films was several orders of magnitude higher on the active papers than on the reference papers and other substrates (Figure 2). Moreover, the AgNP film resistivity on the active papers increased significantly on coatings with larger pore sizes (Figure 2a, solid curves). The resistivity of the films on the active paper with the largest pore size (HP22) was 300,000 times higher than the resistivity of bulk silver (RBS, RBS = 1.59 × 10^−8^ Ωm) after treatment at 60 °C. In contrast, the films on the reference papers had a low resistivity of 10–17 RBS already at 60 °C, which decreased to 5–7 RBS at 180 °C (Figure 2a, dashed curves).

**Figure 3:**
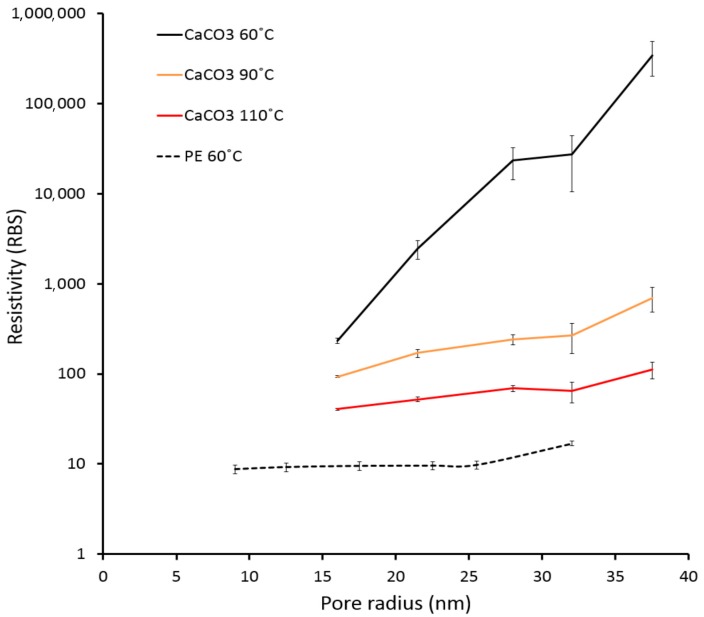
Resistivity of AgNP films as a function of the coating pore radius. The sintering inhibition on active papers (solid curves) is stronger when using larger pore sizes. The error bars correspond to ±1 standard deviation. RBS = resistivity of bulk silver.

Adding chloride to the reference papers before printing increased the resistivity of the AgNP films. Pretreating a HP16 reference paper with 0.1-M HCl increased the resistivity from 10 RBS to 19 RBS at 60 °C (Figure 2b). The AgNP films printed on commercial comparison substrates (PET film and LWC paper) had comparably low resistivities of 26 RBS and 14 RBS, respectively, after treatment at 60 °C (Figure 2b). We conclude that the active paper series induced significant sintering inhibition compared to other substrates. Further, increasing the amount of chloride increased the resistivity on the reference series, thus indicating that chloride is involved in the sintering inhibition. The sintering inhibition was much stronger when using coatings with larger pore sizes (Figure 3).

### 2.3. Chloride Migrated from the Coating into the AgNP Film

**Figure 4:**
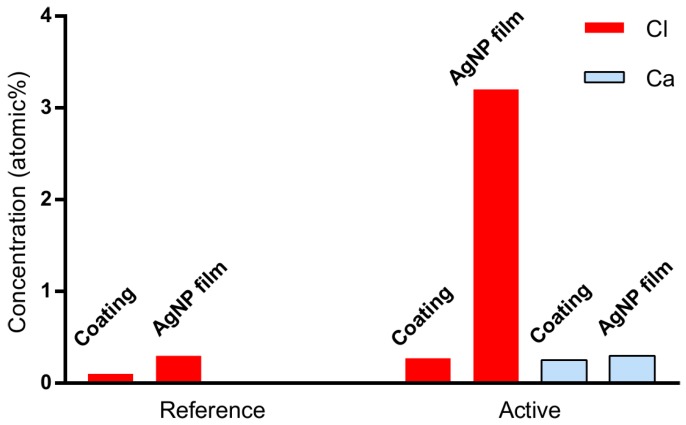
Near-surface concentrations of Cl (red bars) and Ca (blue bars) in the HP14 coating (measured with SEM-energy-dispersive X-ray spectroscopy (EDS)) compared with the surface concentrations in AgNP films printed on the same coating (measured with X-ray photoelectron spectroscopy (XPS) surface element analysis). The concentration of Cl in the AgNP film is much higher than that in the coating, suggesting that Cl^−^ is injected into the AgNP film during the absorption of the ink solvent and the formation of the AgNP film. The results for the other coatings (HP10–HP22) were similar.

We analyzed the elemental concentration in the paper coatings and corresponding AgNP films and found significant migration of chloride into the AgNP films on the active papers. This supports the hypothesis that chloride is involved in the sintering inhibition. The active papers had near-surface element concentrations of Cl and Ca close to 0.3 atomic %, whereas the reference papers had a Cl concentration of 0.1 atomic % and no detectable Ca. To examine the extent to which Cl and Ca interact with the AgNP ink during deposition and drying, we performed X-ray photoelectron spectroscopy (XPS) surface element analysis of the AgNP films after inkjet printing. On the active papers, the concentration of Cl in the AgNP films was 3.2 atomic %, a 10-fold increase compared to the 0.3 atomic % Cl in the coating (Figure 4). In contrast, the concentration of Ca did not increase, exhibiting similar levels in the AgNP film and the coating. On the reference papers, the concentration of Cl in the AgNP films was 0.3 atomic %, which corresponds to a 3-fold increase compared to the 0.1 atomic % concentration of Cl in the coating (Figure 4). Therefore, we conclude that Cl^−^ was injected into the AgNP film from the coating during the ink absorption and film setting process, increasing its concentration. The extent of the Cl^−^ transfer was much larger on the active paper (3.2 atomic % Cl on top of the AgNP film) versus on the reference paper (0.3 atomic % Cl on top of the AgNP film).

### 2.4. Chloride Impaired the Colloidal Stability of the AgNP Ink

Flocculation tests showed that Cl^−^ impaired the colloidal stability of the AgNP ink, indicating that Cl^−^ should facilitate sintering rather than inhibit it. Flocculation tests of diluted ink dispersions (0.002 wt % Ag) showed that CaCl_2_ (0.01 M and 0.1 M) impaired the stability of the dispersion. In contrast, the presence of various other salts (CaCO_3_, Na_2_CO_3_, and NaHCO_3_ at concentrations of 0.01 M and 0.1 M) did not affect the colloidal stability. The PVP capping agent used for these AgNPs is a nonionic polymer. Thus, the stabilization mechanism is mainly steric, effective over a wide pH range, and insensitive to charges [15]. Further, we confirmed experimentally that the AgNP dispersion was stable over a wide pH range of 2.4–12. Therefore, the observed destabilization in the presence of CaCl_2_ indicates that Cl^−^ desorbs the PVP capping agent from the AgNPs. In this case, it follows that Cl^−^ should act as a sintering-assisting agent. Experimental studies [10,11,12,13] have indeed demonstrated assisted sintering resulting from capping agent desorption in the presence of Cl^−^. For example, Olkkonen et al. [16] reported the assisted sintering of a AgNP ink by exposing inkjet-printed films to water containing NaCl and the authors argued that the presence of Cl^−^ in the water increased the probability of polymer desorption. We conclude that our results of Cl^−^ induced sintering inhibition directly contradict the previous studies of Cl^−^ induced sintering of AgNP films, which is interesting. The unexpected resistivity increase of AgNP films on active papers suggests that Cl^−^ must be involved in a mechanism that counters the effect of capping agent desorption and yields a net effect of sintering inhibition. The results indicate that this mechanism may be in-film salt formation and therefore dependent on the availability of Cl^−^ during the AgNP film formation and drying processes.

### 2.5. Chloride Had Opposing Effects on Room-Temperature Sintering: Pretreatment vs. Post-Treatment

**Figure 5:**
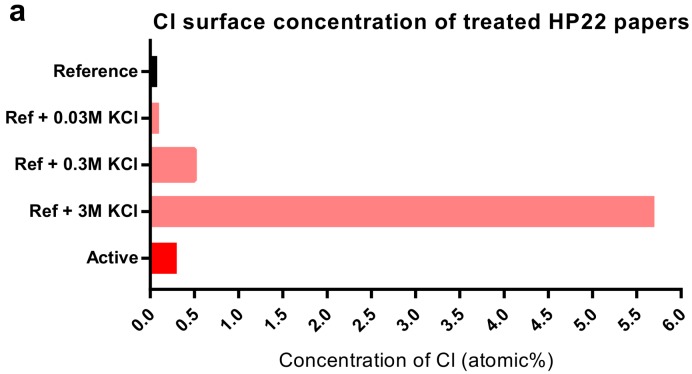

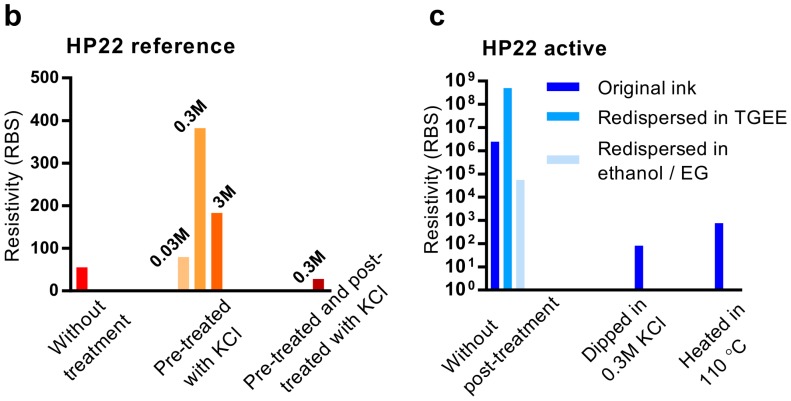
The impact of KCl pretreatment vs. KCl post-treatment on AgNP film resistivity, after drying in room temperature for 48 h. (**a**) Near-surface concentrations of Cl in the untreated and pretreated HP22 papers (EDS). (**b**) The impact of KCl pretreatment/post-treatment using reference papers. KCl pretreatment increased the resistivity, whereas KCl post-treatment reduced the resistivity. (**c**) The impact of the ink solvent and post-treatment using active papers. The choice of the ink solvent affected the resistivity, but it remained high for all tested solvents. Dipping the films in 0.3 M KCl at room temperature sintered the films more effectively than heating at 110 °C for 5 min. Units in RBS (resistivity of bulk silver).

The HP22 active papers had the strongest sintering inhibition effect (Figure 2a) and we therefore selected the HP22 active papers and corresponding reference papers for further experiments. We prepared three modified HP22 reference papers with increased Cl concentrations by pretreating them with 0.03 M, 0.3 M, and 3 M KCl, respectively (Figure 5a). The KCl pretreatment of reference papers increased the resistivity of AgNP films. The resistivity was 55 RBS on the untreated reference paper but after KCl pretreatment the reference papers exhibited higher resistivities, with a maximum of 380 RBS for the 0.3 M KCl pretreatment (Figure 5b). However, dipping the AgNP films in KCl solution had the opposite effect, lowering the resistivity. Dipping the 380-RBS films on pretreated reference paper into 0.3 M KCl for 3 s resulted in film sintering, lowering the resistivity from 380 RBS to 28 RBS (Figure 5b). The AgNP films on active papers were also sintered by dipping them in 0.3 M KCl solution, decreasing the resistivity from 2.5 × 10^6^ RBS to 82 RBS (Figure 5c). In comparison, oven heating the films at 110 °C was less effective at achieving sintering, lowering the resistivity from 2.5 × 10^6^ RBS to 760 RBS (Figure 5c). We conclude that Cl^−^ assisted sintering when the AgNP film was dipped into KCl solution, which is consistent with the expected effect of PVP desorption. Interestingly, however, Cl^−^ were apparently involved in another mechanism, inducing sintering inhibition, when KCl was present in the coating during film formation. To examine if the AgNP capping agent and the ink solvent have a large influence on the observed inhibition effect, we compared the resistivity using the main ink with the resistivity using two other ink variants: (i) Var1 with a different solvent and (ii) Var2 with a different capping agent (Table 1). When changing the ink solvent from TGEE to ethanol/ethylene glycol, the resistivity remained very high (Table 1, Figure 5c). However, using an ink with another capping agent, the resistivity dropped more than four orders of magnitude (Table 1). Therefore, it is indicated that the PVP capping agent facilitates the mechanism of the sintering inhibition.

### 2.6. AgCl Nanocrystal Formation Is THE Main Sintering Inhibition Mechanism

**Figure 6:**
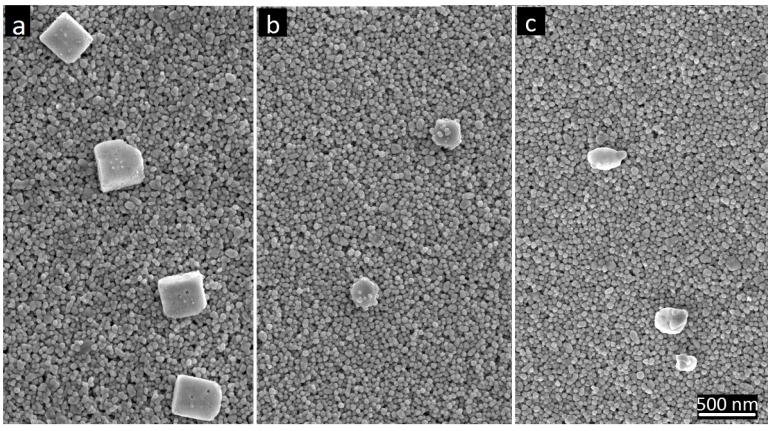
AgCl formation on top of the AgNP films (SEM images). (**a**) HP22 reference (pretreated with 0.3 M KCl), (**b**) HP10 active, and (**c**) HP22 active papers.

**Figure 7:**
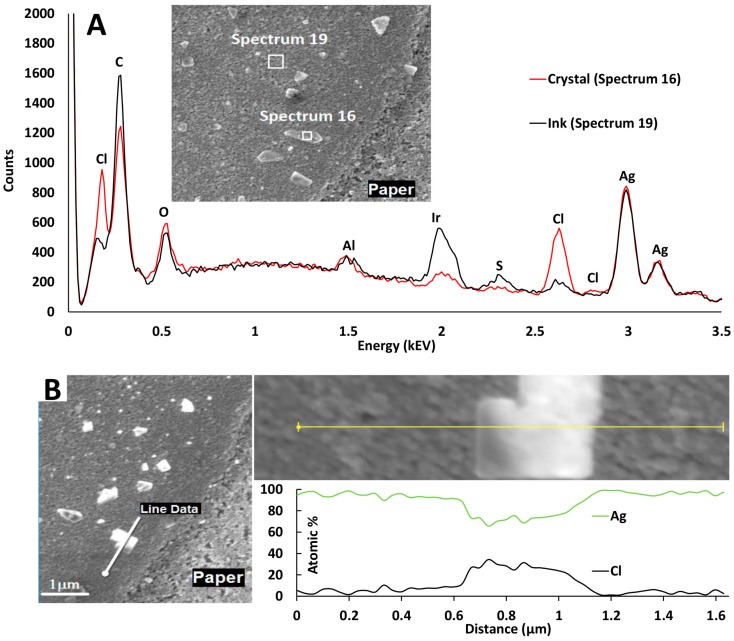
Verification of AgCl composition in crystals (energy dispersive spectroscopy, EDS). (**A**) Surface analysis of a crystal compared with the ink surface reference (scan areas shown as white squares). (**B**) Line analysis across a crystal. The Cl content is measured to approximately 30 atomic % across the crystal (the exact content of 50 atomic % is not reached because the electron beam is partially penetrating through the crystal so that the measurement is affected by the underlying AgNP ink layer).

To explain the main cause of the observed sintering inhibition, we analyzed high-resolution SEM images of AgNP films on active papers and reference papers and observed the formation of nanosized AgCl crystals (Figure 6 and Figure 7 and Figure S1). For the active papers and KCl-pretreated reference papers, AgCl crystals with a size distribution of 80–600 nm formed on top of the AgNP films (Figure 6 and Figure S1). However, on the untreated reference papers, no AgCl formation was observed, likely because of insufficient concentrations of surface Cl. The observed AgCl formation supports the hypothesis from an earlier study [14], in which this AgNP ink exhibited increased resistivity on papers pretreated with NaCl and NaBr, and it was speculated that the precipitation of silver salts could explain the behavior.

**Figure 8:**
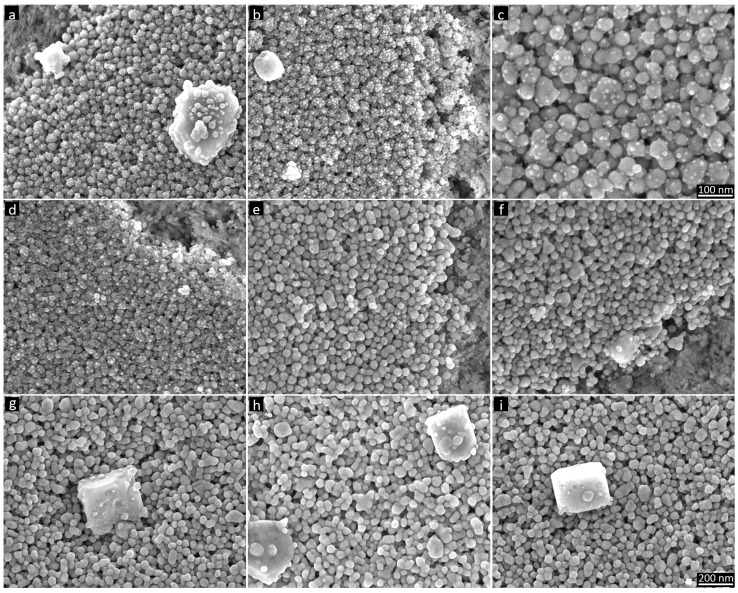
AgNP films on active papers and reference papers showing details of nanocrystal formation (SEM images). (**a**) HP10 active, (**b**) HP22 active, (**c**) HP22 active, (**d**) HP22 active (ethanol + EG), (**e**) HP22 reference, (**f**) HP22 reference (0.3 M KCl pretreatment), (**g**) HP22 reference (0.3 M KCl pretreatment), (**h**) HP22 reference (0.3 M KCl pretreatment + dipping), and (**i**) HP22 active (0.3 M KCl dipping) papers. All images are shown using the same scale, except for the top right image.

On the active papers, additional significant formation of AgCl nanocrystals was observed directly on the surfaces of individual AgNPs (Figure 8a–c). We believe that this particular nanocrystal formation is the main mechanism explaining the high resistivity and inhibited sintering characteristics on the active papers. We propose that the 5–15-nm AgCl nanocrystals reduce the number of direct AgNP-AgNP contact points in the film, replacing them with AgCl bridges. Because AgCl is a semiconductor, this effect greatly reduces the number of effective current percolation paths and increases the film resistivity. The formation of AgCl on the active papers occurred predominantly as nanocrystals on AgNP surfaces, whereas that on the pretreated reference papers produced fewer, larger crystals that exerted much less influence on the AgNP film resistivity. This difference in crystal formation is likely attributable to the different absorption rates resulting from the different precoating types. The porous precoatings on the active papers implied that the ink films were instantly dried, but the nonporous precoatings on the reference papers implied a drying time of several minutes. This slower drying on the reference papers corresponded to longer durations of ion diffusion and Ostwald ripening, resulting in larger AgCl crystal sizes and predominant precipitation on top of the coatings. This finding is also consistent with the results obtained by Chiu et al. [17], who studied AgCl formation and migration in mesoporous thin films. These authors observed the growth of AgCl crystals on the surface of drying mesoporous sol-gel silica films containing AgCl nanocrystals and found that larger film thicknesses resulted in fewer and larger crystals. The authors concluded that AgCl nanocrystals migrated through the pores of the drying and shrinking silica film, forming larger crystals at the film surface via Ostwald ripening.

In this study, a faster absorption promoted the formation of smaller crystals and thereby promoted sintering inhibition. This behavior is consistent with the observation that active paper coatings with larger pore sizes and faster absorption (HP22) resulted in a larger number of AgCl nanocrystals on the AgNP surfaces than the HP10 coating (Figure 8a,b). Moreover, the larger number of AgCl nanocrystals is consistent with the higher resistivity of AgNP films on the HP22 coating (Figure 2a). Using the slower-absorbing KCl-pretreated reference papers, we found no nanocrystals on the surface of individual AgNPs (Figure 8f,g). Instead, larger AgCl crystals on top of the AgNP film and crystals embedded in the film formed (Figure 8f,g). Therefore, the moderately increased resistivity of the AgNP film that resulted from the KCl pretreatment of reference papers is likely attributable to a somewhat different mechanism involving a smaller number of embedded AgCl crystals with larger average sizes.

In contrast to the sintering inhibition that resulted from a coating presence of KCl, dipping the AgNP films in 0.3 M KCl had a sintering effect, which can be seen in Figure 8h as neck formation between NPs. Dipping the high-resistivity AgNP films on active papers in 0.3 M KCl dissolved the AgCl nanocrystals on the AgNP surfaces and resulted in moderate film sintering (Figure 8i). Although the solubility of AgCl is very low, nanosized AgCl crystals have high surface energy, which increases their local solubility. Exposing the AgNP film to a solvent could, therefore, dissolve the nanocrystals and redeposit material onto the surfaces of larger crystals via Ostwald ripening [18]. 

**Figure 9:**
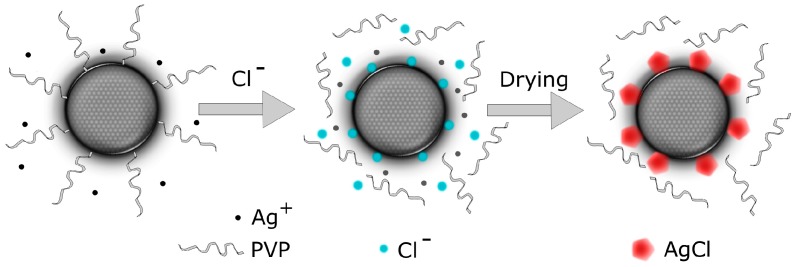
Schematic of proposed AgCl nanocrystal formation on the surface of an AgNP. Chloride ions induce desorption of the PVP capping agents by replacing the PVP anchor group at the AgNP surface. The surface-attached chloride ions act as seeds for AgCl crystals that form under suitable conditions (the presence of free ions, quick absorption/drying).

The AgCl nanocrystals that formed on the surface of the AgNPs presumably grow from chloride ions that attach to the AgNPs during desorption of the PVP capping agent. Magdassi et al. [12] showed that PAA capping agents are adsorbed to AgNP surfaces through Ag-O coordination. They provided direct evidence that the PAA desorbed from the AgNPs in the presence of Cl^−^ and proposed the following desorption mechanism: Cl^−^ successfully compete for the binding sites, replacing the PAA anchor groups with Cl^−^ and thereby detaching the PAA capping agents. Because it has been shown [19,20,21,22] that PVP capping agents also adsorb to Ag nanostructures through Ag-O coordination, we suggest that Cl^−^ desorb the PVP capping agents via the same anchor group-replacement mechanism. The proposed mechanism for the surface AgCl nanocrystal formation is illustrated in Figure 9. When the ink is deposited, the ink solvent wets the substrate and Cl^−^ is injected from the substrate into the wet AgNP film. Then, Cl^−^ desorb the PVP capping agents by replacing the PVP anchor groups, and therefore Cl^−^ remain on the AgNP surface [11,12]. During the ink solvent absorption and drying of the AgNP film, the surface-attached Cl^−^ act as seeds for AgCl nanocrystal formation. These nanocrystals can form if sufficient concentrations of free Ag^+^ and Cl^−^ are present and if the ion-diffusion duration, which is determined by the ink-absorption rate, is suitable.

In summary, the sintering inhibition was due to AgCl crystal formation within the AgNP film. Two important factors affecting the crystal formation were identified: (i) a sufficient substrate concentration of chloride and (ii) quick solvent absorption. Additionally, the capping agent of the AgNPs likely plays an important role in AgCl formation, however this role remains unknown at this stage and need to be studied further. These findings may also have relevance outside the printed electronics field because Ag/AgCl nanocomposites have been shown to possess enhanced photocatalytic and antibacterial properties [23]. In this context, the demonstrated inkjet printing technique can be regarded as a nearly instant synthesis method for Ag/AgCl nanocomposites that is capable of high-resolution patterning. 

## 3. Materials and Methods

### 3.1. Comparison Substrates and Substrate Pretreatments

Two commercially available substrates were used for comparison. (1) Nonporous PET film (Mitsubishi Hostaphan RN, 100 μm): before printing, this film was ultrasonicated in water for 15 min at 60 °C, rinsed with ethanol and distilled water, and dried. (2) LWC paper (SCA LWC GC80, Sundsvall, Sweden): this substrate’s coating consisted of CaCO_3_/kaolin clay with a styrene butadiene binder. No Cl content was detectable in the coating using SEM-EDS. Papers were pretreated by depositing HCl (0.1 M) and KCl (0.03 M, 0.3 M, and 3 M) using the Meyer-rod coating technique with a 10 μm wet film thickness followed by drying at 60 °C.

### 3.2. Inks and Printing

A commercially available AgNP inkjet ink (DGP40LT-15C, Advanced Nano Products, Sejong, Korea) containing 31 wt % AgNPs (40–60 nm), PVP as a capping agent, and TGEE as a solvent was used. The colloidal stability of the AgNP ink at various pH values and in the presence of various salts was examined with a turbidimeter (Hach Ratio XR, Loveland ,USA) according to the following procedure: the ink was diluted with triethylene glycol monomethyl ether (TGME, analytical grade) in a ratio of 1:25. Then, 50 μL of the diluted ink was added to 30 mL of the test solution. Immediately after mixing, the pH of the solution was measured and the turbidity monitored, as a function of time. The ink solvent and deionized water served as reference test solutions. To vary the pH, various concentrations of CH_3_COOH (>99.8%, Fluka) or NaOH (>99%, Riedel-de Haën) were used. The salts tested were CaCO_3_, Na_2_CO_3_, NaHCO_3_, and CaCl_2_ at concentrations of 0.01 M and 0.1 M.

The exchange of ink solvent was achieved by the following procedure: AgNPs from the original AgNP ink were collected by centrifugation (relative centrifugal force RCF = 11,000 *g*, 60 min), washed twice with ethanol, and then dried (35 °C, 60 min). The AgNPs were redispersed into two new inks, each containing 28 wt % AgNP: (1) 0.37 g of AgNPs in 1 mL of TGEE (technical grade, Fluka), and (2) 0.37 g of AgNPs in 0.41 mL of ethanol (99.5%, Solveco) +0.59 mL of EG (99.9%, VWR). The 40/60 volume proportion of ethanol/EG has a surface tension and viscosity very similar to those of TGEE.

A piezoelectric inkjet printer (Dimatix 2831, Santa Clara, CA, USA) was used to print horizontal conductors with nominal dimensions of 20 × 0.4 mm^2^. Ink cartridges with a drop volume of 10 pL (Dimatix 11610) were used with a voltage of 24 V and an optimized waveform supplied by the ink manufacturer. The drop spacing was set to 20 μm. The nozzle and platen temperatures were controlled at 22 °C.

### 3.3. Sintering

Oven sintering was performed by an initial low-temperature treatment at 60 °C for 10 h, followed by stepwise heating to 90, 110, 150, and 180 °C, with 5 min at each temperature. Electrical resistance was measured after each temperature step. The maximum value without conductor failure because of deformation of the polyethylene (PE) precoating was observed at 180 °C. Note that both 150 °C and 180 °C exceeded the melting point of PE and were usable only with careful handling of the PE-precoated reference papers. The CaCO_3_ precoated active papers, LWC paper and PET film resisted normal handling and bending at all temperatures without conductor failure. Room-temperature sintering was performed by dipping AgNP films on paper in 50 mL of 0.3 M KCl for 3 s.

### 3.4. Characterization

The conductor resistance was measured with 4-point probes using a source-meter in current source mode (Keithley 2611A, Solon, OH, USA). To calculate the average value, eight conductors were measured for each substrate. The volume resistivity of each conductor was calculated from the resistance by the equation: R = ρ·L/A, where ρ is the resistivity, R is the resistance, L is the conductor length, and A is the cross-sectional area of the conductor. To calculate the cross-sectional area, the layer thickness was determined by atomic force microscopy (AFM) to be approximately 0.60 μm after sintering. The conductor width and length were 440 μm and 20 mm, respectively, according to measurements performed using a microscope camera. The element concentration measurements were performed by XPS (Kratos AXIS Ultra DLD, Manchester, UK) and EDS (Tescan MAIA3/Oxford Aztec, Brno, Czech Republic). FE-SEM characterization was performed using a Zeiss Merlin system. 

## 4. Conclusions

This work has revealed that chloride can have contradictory roles during low-temperature sintering of AgNP films. Although chloride ions are known to act as chemical agents that assist sintering, we have shown that they can also induce the opposite effect, sintering inhibition due to AgCl nanocrystal formation. These findings reveal that the impact of chloride can be reversed by in-film nanocrystal formation. This new insight is relevant for properly understanding and designing chemical sintering methods.

## Figures and Tables

**Table 1:** Comparison using different inks show that the PVP capping agent has a large influence on the resistivity increase.

Ink	Capping Agent	Solvent	Resistivity(RBS) ^1^
Main	PVP	TGEE	10^6^
Var1	PVP	Ethanol/EG ^3^	10^5^
Var2	Other ^2^	Ethanol/EG ^3^	<100

^1^ HP22 active paper after 48 h drying in room temperature. ^2^ Unspecified by ink manufacturer. ^3^ 40/60 volume ratio of ethanol/ethylene glycol (EG).

## Supplementary Materials

The following are available online at http://www.mdpi.com/2079-4991/7/8/224/s1, Figure S1: SEM images of AgCl formation on top of the AgNP films.
