# Supplementary material for: Sintering Inhibition of Silver Nanoparticle Films via AgCl Nanocrystal Formation
Source: Nanomaterials (Basel). 2017 Aug 17;7(8):224. doi: 10.3390/nano7080224 (PMC5575706; doi:10.3390/nano7080224)
Supplement: Supplementary file 1 [file nanomaterials-07-00224-s001.pdf]

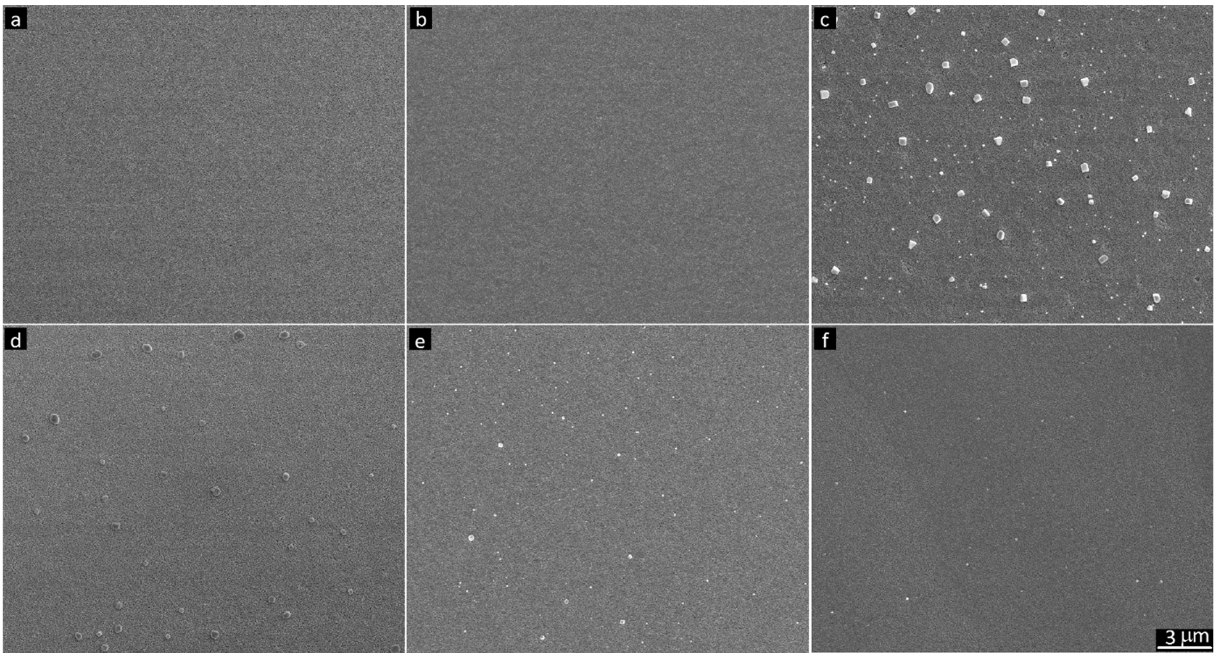

Figure S1. AgCl formation on top of the AgNP films (SEM images). AgCl formation occurred when the AgNP films were printed on paper coatings containing a sufficient concentration of Cl. a) HP10 reference, b) HP22 reference, c) HP22 reference (pretreated with 0.3 M KCl), d) HP10 active, e) HP22 active, and f) HP22 active (ethanol+EG ink) papers.
